# Glutathione S Transferases Polymorphisms Are Independent Prognostic Factors in Lupus Nephritis Treated with Cyclophosphamide

**DOI:** 10.1371/journal.pone.0151696

**Published:** 2016-03-22

**Authors:** Alexandra Audemard-Verger, Nicolas Martin Silva, Céline Verstuyft, Nathalie Costedoat-Chalumeau, Aurélie Hummel, Véronique Le Guern, Karim Sacré, Olivier Meyer, Eric Daugas, Cécile Goujard, Audrey Sultan, Thierry Lobbedez, Lionel Galicier, Jacques Pourrat, Claire Le Hello, Michel Godin, Rémy Morello, Marc Lambert, Eric Hachulla, Philippe Vanhille, Guillaume Queffeulou, Jacky Potier, Jean-Jacques Dion, Pierre Bataille, Dominique Chauveau, Guillaume Moulis, Dominique Farge-Bancel, Pierre Duhaut, Bernadette Saint-Marcoux, Alban Deroux, Jennifer Manuzak, Camille Francès, Olivier Aumaitre, Holy Bezanahary, Laurent Becquemont, Boris Bienvenu

**Affiliations:** 1 Department of Internal Medicine, CHU Caen, Caen, France; 2 Department of Pharmacology, Hôpital Kremlin Bicêtre, Kremlin Bicêtre, France; 3 Department of Internal Medicine, Hôpital Cochin, APHP, Université Paris V, Paris, France; 4 Department of Nephrology, Hôpital Necker, Paris, France; 5 Department of Internal Medicine, Hôpital Bichat, Paris, France; 6 Department of Rheumatology, Hôpital Bichat, Paris, France; 7 Department of Nephrology, Hôpital Bichat, Paris, France; 8 Department of Internal Medicine, Hôpital Kremlin Bicêtre, Kremlin Bicêtre, France; 9 Department of Nephrology, CHU de Caen, Caen, France; 10 Department of Clinical Immuno-pathology, Hôpital Saint Louis, Paris, France; 11 Department of Nephrology and Clinical Immunology, CHU Toulouse, Toulouse, France; 12 Department of Vascular Medicine, CHU Caen, Caen, France; 13 Department of Nephrology, CHU Rouen, Rouen, France; 14 Biostatistics Unity, CHU Caen, Caen, France; 15 Department of Internal Medicine, CHU Lille, Lille, France; 16 Department of Nephrology, CH Valenciennes, Valenciennes, France; 17 Department of Nephrology, CH Cherbourg, Cherbourg, France; 18 Department of Nephrology, CH Charleville Mézière, Charleville Mézière, France; 19 Department of Nephrology, CH Boulogne-sur-Mer, Boulogne-sur-Mer, France; 20 Department of Internal Medicine, CHU Toulouse, France; UMR 1027 Inserm-Univeristy of Toulouse, France; CIC 1436, Toulouse, France; 21 Department of Internal Medicine, Hôpital Saint Louis, Paris, France; 22 Department of Internal Medicine, CHU Amiens, Amiens, France; 23 Department of Rheumatology, Hôpital Aulnay-sous-bois, Aulnay-sous-bois, France; 24 Department of Internal Medicine, CHU Grenoble, Grenoble, France; 25 Department of Immunology, Institut Cochin, Paris, France; 26 Department of Dermatology, Hôpital Tenon, Paris, France; 27 Department of Internal Medicine, CHU Clermont-Ferrand, Clermont-Ferrand, France; 28 Department of Internal Medicine, CHU Limoges, Limoges, France; Instituto Nacional de Ciencias Medicas y Nutricion Salvador Zubiran, MEXICO

## Abstract

**Objective:**

To investigate association between genetic polymorphisms of GST, CYP and renal outcome or occurrence of adverse drug reactions (ADRs) in lupus nephritis (LN) treated with cyclophosphamide (CYC). CYC, as a pro-drug, requires bioactivation through multiple hepatic cytochrome P450s and glutathione S transferases (GST).

**Methods:**

We carried out a multicentric retrospective study including 70 patients with proliferative LN treated with CYC. Patients were genotyped for polymorphisms of the CYP2B6, CYP2C19, GSTP1, GSTM1 and GSTT1 genes. Complete remission (CR) was defined as proteinuria ≤0.33g/day and serum creatinine ≤124 µmol/l. Partial remission (PR) was defined as proteinuria ≤1.5g/day with a 50% decrease of the baseline proteinuria value and serum creatinine no greater than 25% above baseline.

**Results:**

Most patients were women (84%) and 77% were Caucasian. The mean age at LN diagnosis was 41 ± 10 years. The frequency of patients carrying the GST null genotype *GSTT1-*, *GSTM1-*, and the *Ile→105Val GSTP1* genotype were respectively 38%, 60% and 44%. In multivariate analysis, the *Ile→105Val GSTP1* genotype was an independent factor of poor renal outcome (achievement of CR or PR) (OR = 5.01 95% CI [1.02–24.51]) and the sole factor that influenced occurrence of ADRs was the *GSTM1* null genotype (OR = 3.34 95% CI [1.064–10.58]). No association between polymorphisms of cytochrome P450s gene and efficacy or ADRs was observed.

**Conclusion:**

This study suggests that GST polymorphisms highly impact renal outcome and occurrence of ADRs related to CYC in LN patients.

## Introduction

Systemic lupus erythematosus (SLE) is an autoimmune disease that particularly affects young women with a prevalence of 50-150/100,000 in Caucasians [1,2]. Renal involvement is frequent, from 30–74%, depending on the study and the definition of lupus nephritis (LN) and strongly impact prognosis [3,4,5]. Clinical trials have shown that intravenous (IV) CYC, an alkylating agent with a low therapeutic index, is effective in achieving remission and preserving renal function in proliferative LN [6,7]. However, between 30–40% of patients treated with CYC fail to achieve renal remission and response to CYC treatment is difficult to predict [6,7]. The pharmacokinetics and metabolism of CYC have been much studied [8]. As a prodrug, CYC requires bioactivation through multiple hepatic cytochrome P450s (CYP2B6, CYP2C19) to form 4-hydroxy-CYC (4-OH-CYC), which is finally converted to cytotoxic alkylating phosphoramide mustard [9]. Phosphoramide mustard is the therapeutically active metabolite while acrolein is responsible for toxicity. Additionally, 4-OH-CYC is further conjugated with intracellular glutathione by multiple glutathione S transferases (GSTM1, GSTP1, and GSTT1), producing non-toxic 4-glutathionyl-CYC.

Several polymorphisms of CYP2C19 are known to be associated with reduced enzyme activity, among these are CYP2C19*2 characterized by a 681G→A substitution in exon 5, and CYP2C19*3, leading to a stop codon [10]. Carriers of one allelic variant CYP2C19*2 or CYP2C19*3 alleles are considered to have a poor metabolizers (PM) phenotype while homozygous carriers of CYP2C19*1 allele (wild-type allele) are classified as extensive metabolizers (EM). On the other hand, patients presenting CYP2C19*17 allele are considered as ultrarapid metabolizers (UM) [11]. Polymorphisms of CYP2B6 have also been described, patients with CYP2B6*5 or CYP2B6*6 allele are considered as PM compared to the wild-type allele (CYP2B6*1) [12,13]. Thus, PM patients could present a poor response to CYC although UM patients could have an enhanced response to CYC linked to wide inter-patient variability upon exposure to CYC. Deletions in GSTs (GSTT1, GSTM1 and GSTP1) lead to reduction in detoxification enzymatic activity and prolonged exposure to CYC with increased risk of ADRs (adverse drug reactions) but also lead to the possibility of improved response [14].

Therefore, in this study, we assessed the hypothesis that genetic polymorphisms of GSTs or CYP could impact remission and ADRs related to CYC in LN patients.

## Patients and Methods

We carried out a multicentric retrospective study in France.

### Patients

Patients with biopsy-proven proliferative LN (World Health Organization WHO class III or IV) who were referred to French hospitals and had been treated with CYC pulses before 2006 were identified. Patients included in the “PLUS” study were also screened for eligibility. The diagnosis of SLE in patients was confirmed based on the American College of Rheumatology (ACR) criteria published in 1997. All patients provided written informed consent. This survey was conducted in compliance with the protocol of Good Clinical Practices and Declaration of Helsinki principles and was carried out with the approval of the Regional Ethics Committee Caen.

### Data collection

Clinical, biological data were retrospectively collected. Data were collected from charts using a standarized form that included the following information: gender, month/year of birth, date of first symptoms and diagnosis, clinical and biological lupus manifestations, histological data of renal biopsy, significant comorbidities and ADRs.

### Primary endpoint

Complete remission (CR) was defined as proteinuria ≤0.33g/day and serum creatinine ≤124 μmol/l. Partial remission (PR) was defined as proteinuria ≤1.5g/day with a 50% decrease of the baseline proteinuria value and serum creatinine no greater than 25% above baseline, at the 12^th^ month after the first CYC infusion. Global remission (GR) was calculated by identifying all patients with either CR or PR.

### DNA extraction and cytochrome P450/*GST* genotyping

Salivary DNA samples were collected prospectively from each of patient, except for the patients included in “PLUS” study for who the blood DNA samples were already collected. DNA was extracted from salivary samples using the Puregene DNA Isolation kit (Puregene DNA isolation Kit; Merck Eurolab, Lyon, France), according to the manufacturer’s instructions. Genotyping was performed with the Taqman allelic discrimination technique on an ABI Prism 7000 (TaqMan®) as previously described [15]. Genotyping for common variant alleles of the CYP2B6 gene [CYP2B6*5(1459C>T, rs3211371), CYP2B6*6 (G516T, rs3745274 and A785G, rs3745274)], CYP2C19 gene [CYP2C19*2 (681G>A, rs4244285), CYP2C19*3 (636G>A, rs4986893), CYP2C19*7 (806C>T, rs12248560)]. *GSTM1* and *GSTT1* null mutations were analyzed by a polymerase chain reaction (PCR)-multiplex procedure. This technique clearly identifies the homozygous null genotype but does not discriminate the deletional heterozygotes from non deletional homozygotes, both of which were classified as GSTM1 and T1 positive genotype (*GSTM1+*, *GSTT1+)* or GSTM1 and T1 null genotype (*GSTM1-*, *GSTT1-)* [16]. The GSTP1 codon 105 polymorphism (Ile→Val; C.31A>G) was analyzed by a PCR-restriction fragment length polymorphism (RFLP) assay

### Statistical analysis

Descriptive statistics used included the mean (SD) as appropriate for continuous variables, and frequency (percentage) for categorical variables. Univariate analysis used included the chi-square or Fisher's exact test as appropriate to compare categorical variables and the non-parametric Mann-Whitney test to compare continuous variables. Multivariate analyses were performed with logistic regression. Efficacy was reported by treatment period. Statistical analyses were performed using EpiData^TM^ (EpiData Software version 2.0, "The EpiData Association" Odense, Danemark).

## Results

### Patient characteristics

The clinical and biological characteristics of the 70 patients included in this study at diagnosis of LN are shown in Table 1. Most patients were women (female/male ratio 5.36) and on the 26 patients whom ethnic origin was analysed 77% were Caucasian. The mean age was 41 ± 10 years. All patients carried anti-DNA antibodies. The mean glomerular filtration rate (GFR) was 66 ± 33 ml/min/1.73m^2^. Eighty percent of the patients presented with a class IV WHO LN. All received IV pulses of CYC in first line and the cumulative dose of CYC was 6.2 ± 2.9 g. Eight patients were treated with low dose of CYC (6 pulses of 500 mg) according to the “Eurolupus” schedule. All patients received corticosteroids. Forty percent have also been treated with an angiotensin-converting enzyme inhibitor and 68.6% had received hydroxychloroquine.

**Table 1:** Patient characteristics at baseline.

**Clinical characteristics at baseline**	
Female/Male Ratio, n	5.36 (59/11)
Ethnic origin, caucasian, n, %	20/26 (77)
Ethnic origin, african, n, %	3/26 (11.5)
Ethnic origin, asiatic, n, %	3/26 (11.5)
Age at diagnosis of LN, years, mean, SD	41 ± 10 [23–63]
**Biological/Histological characteristics of LN**	
GFR (MDRD), ml/min/1,73m^2^, mean, SD	66.64 ± 32.99 [18–187]
Creatinine, μmol/l, mean, SD	101.10 ± 57.22 [40–340]
Proteinuria, g/l, mean, SD	3.25 ±3.22 [0–17]
Positive Anti-DNA antibodies, n, %	70/70 (100)
Class III WHO nephritis, n, %	14/70 (20)
Class IV WHO nephritis, n, %	56/70 (80)
**Treatment of LN**	
Cumulative dose of CYC, g, mean, SD	6.23 ± 2.98 [1.,8–16.2]
CYC pulses, mean, SD	7.0 ± 2.3 [3–13]
Patients treated according to “EUROLUPUS” schedule, n, %	8/70 (11.4)
Angiotensin-converting enzyme inhibitors, n, %	28/70 (40)
Hydroxychloroquine, n, %	48/70 (68.5)

LN (Lupu Nephritis); GFR (Glomerular Filtration Rate); WHO (World Health Organization)

### Efficacy and ADRs related to CYC

CR, PR and GR rates at the 12^th^ month after first CYC infusion or during the first 12 months are indicated in Table 2. The GR rate at the 12^th^ month after first CYC infusion was 79%; 58% of patients experienced CR and 42% PR. Thirty four percent of the patients presented at least one ADR related to CYC. ADRs are detailed in Table 2. The most frequently experienced ADRs were nausea and/or vomiting (50%).

**Table 2:** Efficacy and ADRs related to CYC.

**Efficacy of CYC**	
Global remission at the 12^th^ month	78.9% (45/57)
Partial remission at the 12^th^ month	57.7% (26/45)
Complete remission at the 12^th^ month	42.2% (19/45)
Global remisson during the 12^th^ month	81.8% (54/66)
**Adverse drug reactions (ADRs)**	
Patients displaying ARDs	34.3% (24/70)
Nausea	50% (12/24)
Neutropenia	8.3% (2/24)
Infection	8.3% (2/24)
Rash	25% (6/24)
Amenorrhea,	20.8% (5/24)
Diarrhea	8.4% (2/24)

CYC (Cyclophosphamide)

### Study population and allele frequencies

For the entire study population, the observed allele frequencies for each enzyme were in Hardy-Weinberg equilibrium and there is no absence of linkage disequilibrium for CYP or GST variants. The CYP2C19*2 allele was found in 33% of the study population, with 3 homozygous CYP2C19*2 alleles. One patient carried the heterozygous CYP2C19*3 allele. The CYP2C19*17 allele was found in 35% of the population, with 3 homozygous alleles. The CYP2B6*5 allele was found in 17% and the frequency of the CYP2B6*6 allele was 60% with 9 homozygous alleles. The frequency of patients carrying the deficient allele *GSTT1 (GSTT1)*, *GSTM1(GSTM1-)*, GSTP1 p.Ile105Val allele, were 37.7%, 59.4% and 44.3%, respectively. All results are consistent with previous studies on healthy individuals [13, 14] or LN populations [17].

### Association between allele frequencies and renal remission induced by CYC

The global response to CYC during the first 12 months of treatment according to the genetic polymorphisms of CYP2B6, 2C19 and GST are illustrated in Tables 3 and 4. In univariate analysis, the polymorphism of GST, CYP2B6 and CYP2C19 did not influence the efficacy of CYC, except the *Ile→105Val GSTP1*genotype, which showed a trend toward a lower probability of achieving GR (72.4% versus 91.2%, p = 0.059). CR or PR at month 12 were not influenced by the polymorphisms of the GST, CYP 2B6 and CYP2C19 (data not shown).

**Table 3:** Association between polymorphism of CYP2C19 and CYP2B6 with renal remission and ADRs.

	Genotype	n	Global remission % (n)	ADRs %(n)
**Ultrarapid metabolizer**	CYP2C19*17/*1	15	78.6% (11/14)	26.7% (4/15)
	CYP2C19*17/*17	3	100% (3)	0% (0/3)
**Extensive metabolizer** (WT)	CYP2C19*1/*1	23	78.2% (18/23)	30.4% (7/23)
	CYP2B6*1/*1	18	83.3% (15/18)	45% (9/20)
**Poor metabolizer**	CYP2C19*2/*1	15	84.6% (11/13)	40% (6/15)
	CYP2C19*2/*2	3	66.6% (2/3)	66.7% (2/3)
	CYP2C19*3/*1	1	100% (1/1)	0% (0/1)
	CYP2C19 *3/*3	0	NA	NA
	CYP2C19*2/*3	0	NA	NA
	CYP2B6*5/*1	7	85.7% (6/7)	14.2% (1/7)
	CYP2B6*5/*5	0	NA	NA
	CYP2B6*6/*1	28	77.8% (21/27)	32.1% (9/28)
	CYP2B6*6/*6	8	87.5% (6/7)	25% (2/8)
	CYP2B6*5/*6	4	75% (3/4)	50% (2/4)

ADRs (Adverse drug reaction); WT (Wild-type allele); NA (Not applicable)

**Table 4:** Association between polymorphism of GST with renal remission and ADRs.

	Genotype	n	Global remission % (n)	ADRs % (n)
**Extensive metabolizer**	*GSTT1+*	53	82.7% (43/52)	37.7% (20/53)
	*GSTM1+*	28	73% (19/26)	21.4% (6/28)
	*GSTP1 C*.*313A*	36	91.2 (31/34)	30.6% (11/36)
**Poor metabolizer**	*GSTT1-*	16	76.9% (10/13)	25% (4/16)
	*GSTM1-*	41	87.1% (34/39)	43.9% (18/41)
	*GSTP1 C*.*313A>G*	31	72.4% (21/29)	35.5% (11/31)

*ADRs (Adverse drug reaction);* GSTT1+ (*wild-type allele) or - (null allele);* GSTM1+ (*wild-type allele) or - (null allele);* GSTP1 C.313A (*wild-type allele) or* GSTP1 *C*.*313A>G (Ile->105Val; homozygotes and heterozygotes combined)*.

### Association between genotype frequencies and ADRs related to CYC

Association between ADRs and genetic polymorphisms of CYP2B6, CYP2C19 and GST are summarized in Tables 3 and 4. The polymorphisms of the GSTT1, GSTP1, CYP2B6 and CYP2C19 genes did not influence occurrence of ADRs. Only the deficient *GSTM1* allele (*GSTM1-*) was associated with more ADRs than the *GSTM1* wild-type allele (*GSTM1*+): 43.9% (18/41) versus 21.4%, (6/28); p = 0.046 in univariate analysis.

### Univariate and multivariate analyses of variables associated with the achievement of global remission and ADRs related to CYC

Several bio-clinical variables associated with the achievement of GR at the 12^th^ month after the first CYC infusion are illustrated in Table 5. None of these variables, including the variable reflecting LN severity (mean GFR, proteinuria, LN histological class) or LN treatment (cumulative dose of CYC or corticosteroids, angiotensin-converting enzyme inhibitors or hydroxychloroquine treatment) influenced the achievement of GR. In multivariate analysis, the *Ile→105Val GSTP1*genotype was an independent factor of poor renal outcome (GR) (OR = 5.011 95% CI [1.025–24.510] p = 0.047).

**Table 5:** Univariate analysis of variables associated with the achievement of global remission after CYC and ADRs.

	Global remission (GR)	Absence of GR	p =
Sex, female, %	25	14	0.385
Age, years, mean	38.54	41.64	0.340
GFR (MDRD), ml/min/1.73m^2^, mean	58.92	65.12	0.22
Proteinuria, g/l, mean	2.59	3.19	0.57
WHO LN class (III versus IV), %	25 vs 75	17 vs 82	0.560
Pulses of corticosteroïds, %,	83.3	65	0.200
Corticosteroïd dosage at 6 months, mg, mean	17.5	20.0	0.499
Angiotensin-converting enzyme inhibitors, %	41.7	37.5	0.529
Hydroxychloroquine, %, mean	45.4	60	0.295
Cumulative dose of CYC, grammes	7.32	5.62	0.081
	**ADRs**	**No ADRs**	**p =**
Sex, male, %	8.3	20	0.182
Age, mean, years	43.2	40	0.962
GFR (MDRD), ml/min/1.73m^2^, mean	66.13	66.93	0.846
Cumulative dose of CYC, grammes, mean	7.75	5.39	< 0.025

LN (Lupus Nephritis); GFR (Glomerular Filtration Rate); WHO (World Health Organization); ARDs(Adverse reactions drug)

Variables associated with occurrence of ADRs are illustrated in Table 5. In univariate analysis, the cumulative dose of CYC influenced occurrence of ADRs: patients with ADRs received 7.75g versus 5.39g in the group without ADRs (p<0.025). In multivariate analysis, the sole variable that influenced the occurrence of ADRs was the polymorphism of GSTM1 (OR = 3.345 95% CI [1.064–10.577] p = 0.039).

## Discussion

Our study attempted to test possible prognostic factors of therapeutic response and ADRs related to CYC treatment in LN by investigating genetic polymorphisms of cytochrome P450s and GST. The observed frequency of the polymorphisms of CYP2C19, CYP2B6 and GST were as expected in a predominantly white population of healthy subjects or LN patients, as shown in Table 3. In this study, the polymorphisms of CYP2C19, CYP2B6 did not influence the response to CYC or the occurrence of ADRs. However, the small sample size of this cohort did not allow subgroup analyses concerning the impact of the homozygous deficient allele. Previously, two studies demonstrated that among LN individuals the CYP2C19*2 deficient allele was associated with lower ovarian insufficiency [17, 18]. Others ADRs were not investigated in these studies. Premature ovarian failure was defined as sustained amenorrhea occurring before 45 years. Our study follow-up period was too short (10.5 years) to detect such long-term ADRs and this ADR was difficult to collect in a retrospective study. Concerning the absence of relationship between polymorphisms of cytochrome P450s and efficacy, our data are consistent with the report of Winoto et al., who analysed 36 patients with LN treated with CYC and showed that there was no correlation between remission and genetic polymorphisms [19].

GSTs are one of the key enzymes that regulate the conversion of toxic compounds to hydrophilic metabolites for the purpose of detoxification. Because of its critical detoxifying role, deficiency in GST enzyme activity due to genetic polymorphisms could attenuate the ability to eliminate CYC and its toxic metabolites which are substrates for GST. Therefore patients carrying *GST* null genotypes could predispose patients to ADRs but also simultaneously undergo enhanced clinical response. Our study demonstrated an association between the *GSTM1* null genotype and ADRs related to CYC: 43.9% versus 21.4%; p<0.05 in univariate analysis. Furthermore, multivariate analysis taking into account age, gender, GFR, cumulative dose of CYC showed that the *GSTM1* null genotype was an independent determinant of ADRs (OR = 3.345 95%CI [1.064–10.577] p<0.05). This report is consistent with previous studies that showed that patients with the *GSTM1-* experienced more toxicity from chemotherapy than patients without this mutation [20]. Concerning SLE, Zhong et al. observed that the *GSTP1* codon 105 polymorphism significantly increased the risks of short-term ADRs, including myelotoxicity and gastro-intestinal toxicity among 102 patients treated with CYC [21]. In this study, the *Ile→105Val GSTP1*genotype had, paradoxically, a trend toward a lower probability of achieving GR in univariate analysis (73.3% versus 91.2%, p = 0.059) and in multivariate analysis *Ile→105Val GSTP1*genotype was an independent factor poor for renal outcome (OR = 5.011 95%CI = [1.025–24.510] p<0.05). Vester et al. have shown that in pediatric nephrotic syndrome treated with CYC, children with *Ile→105Val GSTP1*genotype had a significantly lower rate of sustained remission compared to the wildtype genotype (7 versus 38%, p <0.02) [22]. As GSTP1 is dominantly expressed in the kidney and not in the liver [23], we can speculate that GSTP1 is not mainly involved in CYC metabolism and the lower rate of remission of the *GSTP1* null genotype could be linked with a decreased detoxification in the kidney. Some studies have suggested that reactive oxygen species (ROS) could be implicated in the pathogenesis of lupus [24]. Thus, we can hypothesis that the *Ile→105Val GSTP1* genotype allows ROS to accumulate and induce apoptosis of glomerular cells and thus causes more damage explicating the lower rate of remission.

The main limitations encompass the retrospective nature of the survey and the possible insufficient statistical power to detect small impact of genetic polymorphism of others GST and CYP because only 70 of the 120 patients initially planned were included.

In conclusion, our study showed that polymorphims of the GSTM1 and GSTP1 could impact remission and ADRs in lupus nephritis treated with CYC. Further investigations are clearly warranted to confirm these results, if confirmed, identification of such strong prognostic factors could lead to personalized treatment with an optimized benefit/risk balance in lupus nephritis patients.
